# Endogenous signalling control of cell adhesion by using aptamer functionalized biocompatible hydrogel†
†Electronic supplementary information (ESI) available: Reagents and materials, apparatus and characterization, experimental details and additional data. See DOI: 10.1039/c5sc02565f



**DOI:** 10.1039/c5sc02565f

**Published:** 2015-08-27

**Authors:** Wen Li, Jiasi Wang, Jinsong Ren, Xiaogang Qu

**Affiliations:** a Laboratory of Chemical Biology and State Key Laboratory of Rare Earth Resource Utilization , Changchun Institute of Applied Chemistry , Chinese Academy of Sciences , Changchun , Jilin 130022 , P. R. China . Email: xqu@ciac.ac.cn ; Fax: +86 431 85262656; b University of Chinese Academy of Sciences , Beijing , 100039 , P. R. China

## Abstract

Design of biological signal-responsive biomaterials is essential for controlling cell–cell and cell–matrix interactions. Herein, we developed a dynamic hydrogel to control cell adhesion with biological signals in a cellular microenvironment. The basic principle was based on using nucleic acid aptamer to recognize cell signalling and control the display of bioligands on the hydrogel. Not only exogenous signalling but also endogenous signalling secreted by surrounding cells could activate the dynamic scaffold and tune the cell adhesion state. Since diverse aptamers have been developed, our design can be extended to multiple biological inputs. The biochemical signal-responsive system will greatly enhance the understanding of complex biological processes as well as the ability to manipulate cellular behaviors.

## Abstract 2

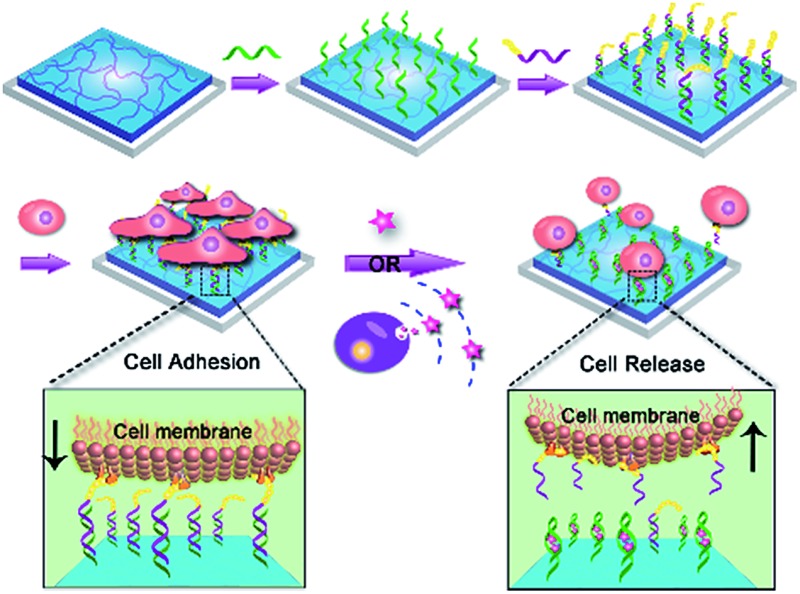
A dynamic hydrogel was fabricated in which the cell adhesion state could be programmed with signaling molecules in a cellular microenvironment.

## Introduction

The fate of a cell is not only regulated by the cell itself but is also highly related to its interactions with the microenvironment.^1^ As the local surrounding of cells, the cellular microenvironment differs from an inert structural support, and is an active component of biochemical signals.^2^ It plays an essential role in regulating cell behavior and even determines the cell fate.^3,4^ Recently, great efforts have been made towards the design of scaffolds to mimic the dynamic features of native cell environments.^5,6^ To this end, some switchable cell scaffolds have been fabricated,^7^ which can controllably tune the components to regulate cell activities. Although interesting, most of the switchable substrates are only able to recognize simple physical or chemical stimuli.^8,9^ In contrast, the cellular microenvironment uses complex biochemical signals to communicate with cells and guide appropriate cell functions.^10^ For example, in vascular regeneration process, neighbouring endothelial cells can secrete certain kinds of growth factor to control the differentiation of stem cells.^11,12^ Similarly. bacteria cells deploy a range of quorum-sensing regulators to communicate with each other and then regulate group behavior.^13^ Therefore, synthetic cell scaffolds with the ability to recognize specific biological inputs are highly desirable.

Engineering materials to control cell adhesion is a fundamental issue for cell biology and medical applications.^14–16^ Cell adhesion not only provides cells an anchoring point to the environment, but also transduces mechanochemical signal into cells.^17,18^ It regulates diverse cell behaviors and physiological events.^19^ In addition, for many biomedical applications, such as tissue engineering and cell-based therapy, it always requires the precise control over when and where cells adhere, migrate, or release.^20,21^ Over the past decade, material science has provided a variety of artificial substrates to control cell adhesion by external stimuli.^22–24^ Most of them are designed to respond to pH,^25^ temperature,^26,27^ magnetic field,^28^ light,^29–31^ or electric field.^32,33^ It should be pointed out that these physicochemical stimuli-activated substrates often could not effectively communicate with real biological systems and the design of biological signal-responsive surfaces to control cell adhesion is still challenging. This is mainly ascribed to the fact that the biological signals are too complex for the synthetic scaffolds to selectively recognize and respond to them. As the current biological recognition systems mainly focus on antigen–antibody interactions, the lack of appropriate screening methods has limited their effectiveness. Additionally, the success in designing one signal-responsive system is often not readily applicable to others, making development in this area very slow.

**Fig. 1:**
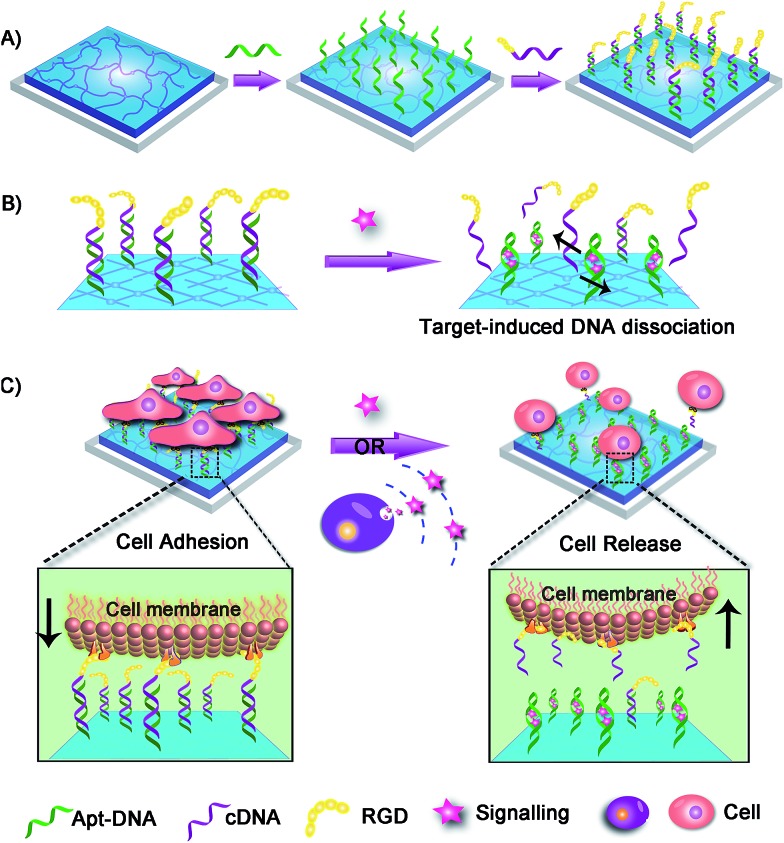
Dynamic control of cell adhesion by using a biological signal-responsive hydrogel. (A) Fabrication of the aptamer-programmed hydrogel. (B) Illustration of the structural change of the DNA scaffold during the target recognition. In the presence of target, aptamer would bind and thereby induce the dissociation of dsDNA. (C) Controlled cell adhesion and release. Both the exogenous and the surrounding cell-secreted signalling could induce structure change of the DNA linker and cause cell detachment.

Herein we fabricated a dynamic hydrogel substrate to regulate cell adhesion with biological signals and the overall concept is illustrated in Fig. 1. Aptamers are single-stranded oligonucleotides that can selectively recognize targets ranging from small compounds, proteins, to whole cells.^34–39^ In comparison to antibodies, aptamers are relatively easy to synthesize, flexible to modification, and stable to denaturation. Recently, aptamers have been successfully used as affinity sites for specific cell capture, cell detection, and targeted therapy.^40–44^ However, they have not been reported for the fabrication of cell microenvironment-mimic scaffolds, which could sense intracellular signalling and thus guide cell fates.

As a proof-of-concept, adenosine triphosphate (ATP) was demonstrated as a signalling molecule here to illustrate the versatility of our strategy. ATP is mainly concentrated in the intracellular cytosol and its extracellular concentration is very low. However, under certain stimulation, ATP will be released from cells to participate in cellular communication and function regulation.^45–47^ The extracellular ATP is therefore regarded as an important signal molecule in biological processes. In our strategy, hydrogel was chosen as the supporting substrate due to its excellent abilities to simulate natural extracellular matrix. The ATP aptamer (Apt-DNA)^48–50^ and its complementary DNA (cDNA) were first conjugated onto the hydrogel. They acted as intelligent mediators to immobilize the cell-adhesive RGD ligand.^51^ The DNA-mediated RGD presentation promoted the adhesion of target cells to the hydrogel surface. However, in the presence of ATP, Apt-DNA would bind to it and form a stable tertiary structure, which promotes the dissociation of the DNA duplex. Subsequently, the original adherent cells would be released form the hydrogel surface. Based on this, the artificial cell scaffold could selectively recognize signalling molecules and then tune the surface properties to regulate cell behavior, which acted in a manner similar to the cell microenvironment. This work will greatly enhance our ability to actively manipulate cellular behaviors in biomedical applications.

## Results

### The fabrication of DNA scaffold-modified hydrogel substrate

**Fig. 2:**
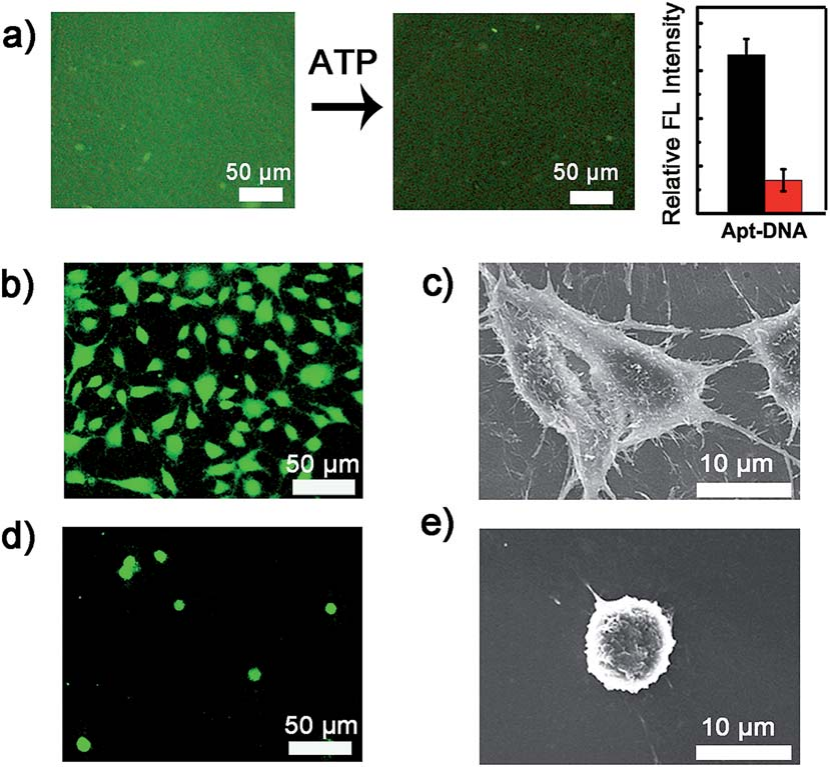
(a) The fluorescence images and the relative fluorescence intensity of FITC-labelled substrates before (left) and after (right) ATP treatment. The fluorescence image (b) and SEM image (c) of cells on the RGD-immobilized hydrogel surface. The fluorescence image (d) and SEM image (e) of cells incubated on the hydrogel without RGD.

The dynamic scaffold was fabricated as shown in Scheme S1 (ESI†). Alginate hydrogel was chosen as the supporting substrate because of its biocompatibility and resistance to nonspecific cell binding. The hydrogel coatings were constructed on glass surfaces *via in situ* photo-polymerization and their formation was monitored by Fourier infrared (FT-IR) spectroscopy (Fig. S1, ESI†). The morphology of hydrogel films was studied by scanning electron micrographs (SEM). As shown in Fig. S2 (ESI†), a uniform film was formed and the surface appeared smooth with no appreciable defects. A fluorescence image of rhodamine B-encapsulated alginate substrate also confirmed the presence and confluency of the hydrogel layer (Fig. S3, ESI†). After the synthesis of hydrogel coatings, the Apt-DNA bearing amine groups were covalently conjugated on the surface through reaction with the carboxyl groups of the alginate polymer. The substrate was then incubated with cDNA to realize hybridization. In previous works, the adenosine-induced aptamer conformational switch and DNA duplex dissociation have been used for design of ATP-responsive sensor, the disassembly of nanoparticles, and controlled drug release.^48–50^ Here, to investigate whether the surface property of hydrogel could be programmed with ATP molecules, we labelled the cDNA with a fluorescent dye, fluorescein isothiocyanate (FITC) (Fig. 2a). After the immobilization of FITC, a bright green fluorescence was observed from the substrate. However, in the presence of ATP molecules, the component change of the hydrogel substrate was visualized by the obvious fluorescence decrease. To confirm that the dimming of fluorescence was caused by ATP-induced aptamer conformational switch, the aptamer-containing double strand DNA (dsDNA) was replaced with a random dsDNA in a control experiment. The ATP treatment had little influence on the fluorescence intensity of the control substrate (Fig. S4, ESI†).

### Cell adhesion on the hydrogel substrate

After fabricating the substrate and confirming its ATP-responsive property, we intended to regulate cell adhesion on the designed substrate. To this end, RGD ligand was modified on the terminus of cDNA to endow the surface with cell adhesive affinity. The hydrogel substrate was then seeded with NIH 3T3 fibroblast cells, to test its cell binding performance. After 3 h of incubation, the cells were stained and characterized with fluorescence microscopy. As illustrated in Fig. 2b, about 1.0 × 10^5^ cells per cm^2^ efficiently attached and spread on the hydrogel surface. The SEM image further showed that the adhered cells displayed fully extended shapes with pseudopodia attached to surface (Fig. 2c). In contrast, only 1.0 × 10^4^ cells per cm^2^ were observed on the control surface that did not present RGD (Fig. 2d). Also cells on the control substrate remained relatively round (Fig. 2e). The differences suggested that alginate hydrogel provided a non-adhesive background and the cells could specially adhere on the hydrogel through the RGD ligand. After culturing for 24 h, the cells could grow well on the hydrogel with high viability (Fig. S5, ESI†) implying the excellent biocompatibility of the hydrogel substrate.

### Controlled cell detachment with exogenous ATP

**Fig. 3:**
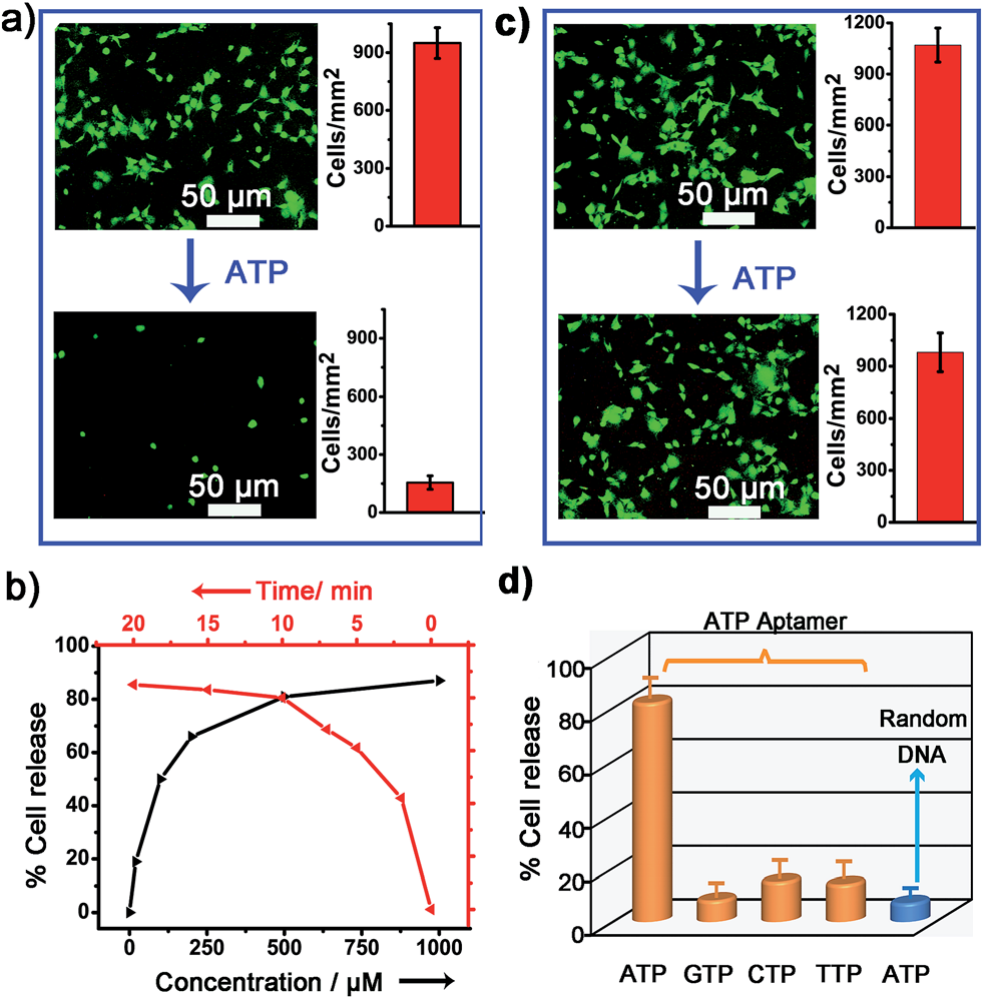
(a) Fluorescence images of ATP-induced cell release on the Apt-DNA based hydrogel substrate. The histograms show the cell number on each surface. (b) The percentage of cells released after exposure to different concentrations of ATP for 10 min (black line) or exposure to 0.5 mM ATP for different times (red line). (c) Fluorescence images and the number of cells on the control substrate before and after ATP treatments. (d) The percentage of cells released on the ATP-, GTP-, CTP- or TTP-treated Apt-DNA hydrogels or on the ATP-treated random DNA hydrogel. The error bars represent the standard deviation of three experiments.

**Fig. 4:**
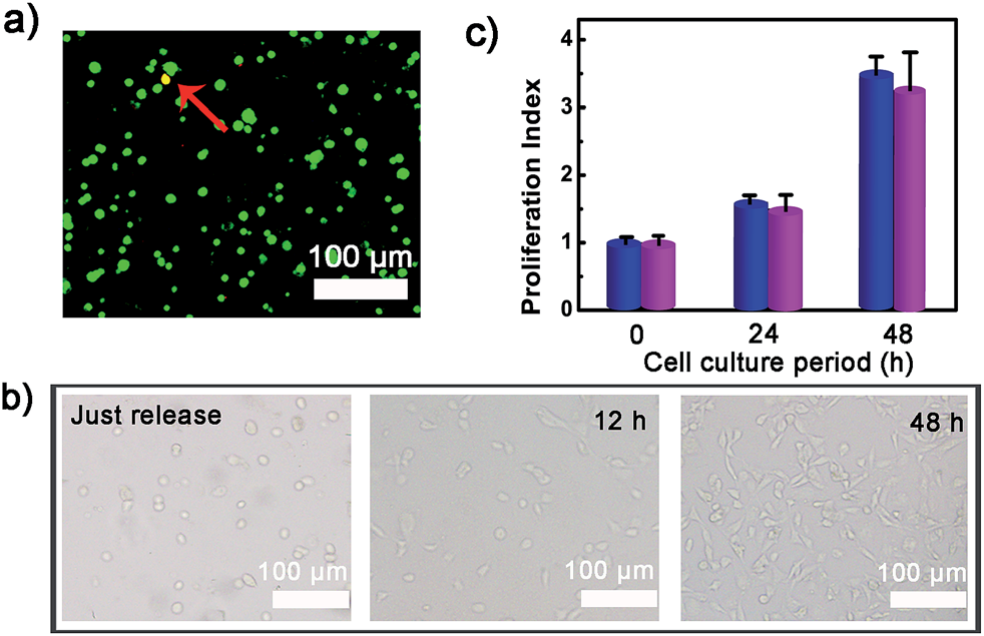
(a) The viability of released cells assessed with a live (green)/dead (red) assay; (b) spreading and proliferation of the released cells after seeding on glass slide for 0, 12 and 48 h; (c) the proliferation ability of normal control cells (blue bar) and released cells (purple bar).

To verify whether the developed surface was applicable to regulate cell adhesion, exogenous ATP was used to stimulate the substrate. Upon incubation with 0.5 mM ATP, most of the original adherent cells became round-shaped and released from the hydrogel substrate (Fig. 3a) and clearly the cell release was ATP concentration-dependent (Fig. 3b). The kinetic study of cell detachment process was also performed. For the random dsDNA-modified control surface, only small amounts of cells were released under ATP stimulus (Fig. 3c). The result was consistent with the fluorescein model assay, which verified that aptamer played a key role in ATP-responsive cell release. It was also noteworthy that cells on the hydrogel surface were not affected by other nucleoside triphosphates including guanosine triphosphate (GTP), cytidine triphosphate (CTP) and thymidine triphosphate (TTP) (Fig. 3d and S6, ESI†). Due to the complexity of the cell environment, the specific recognition was essential for accurate on-demand control of cell adhesion. We then determined the viability of released cells with live/dead assay, which implied that more than 92% of cells were still viable (Fig. 4a). When re-incubated on the glass side, the released cells could grow and proliferate into a confluent layer (Fig. 4b). Their proliferation potential was similar to that of the control population (Fig. 4c and S7, ESI†). These results clearly indicated that the aptamer-based control process did not affect cell viability and the released cells could be used for further biological applications.

### Controlled cell release with ATP secreted from surrounding sender cells

**Fig. 5:**
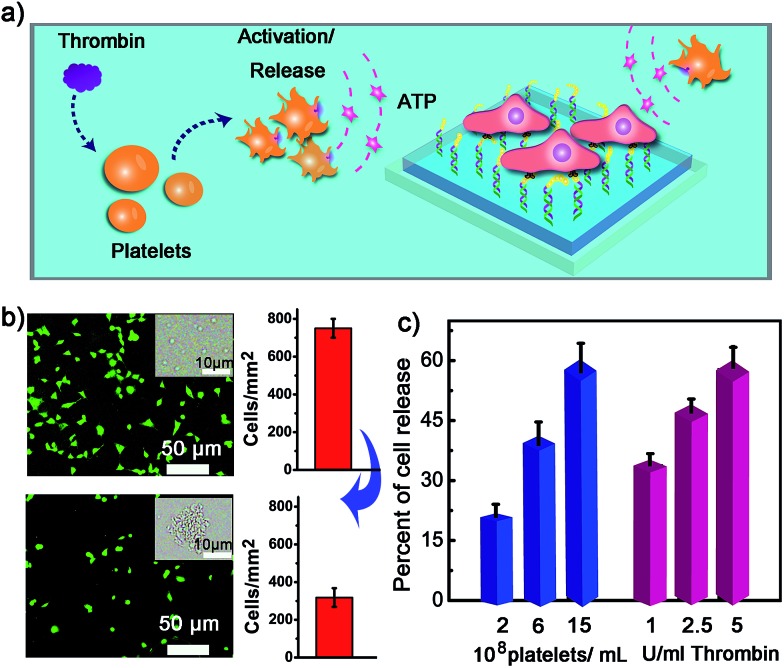
(a) The control of cell adhesion with adenosine molecules released from thrombin-activated platelets. (b) The fluorescence images and the number of NIH 3T3 before (up) and after (below) the activation of platelets by thrombin. Insets show bright field images of platelets. After the addition of thrombin, platelets were activated and formed aggregates. (c) Percentage of NIH 3T3 cell released with the number of platelets co-cultured and the amount of thrombin added for platelet activation.

We next examined whether the adhesion state of cells could respond to ATP molecules secreted from neighboring cells. In cellular microenvironments, cells communicate with each other and the cellular matrix by processing various biological signals, which can further guide cell behavior. ATP is an important signalling agent, and it can be released from cells to regulate neuronal networks, tissue blood flow, tumourigenesis, and host–pathogen interactions. Here, platelets were used as the model senders of endogenous ATP since their ATP release process has been clearly demonstrated (Fig. 5a). When the platelets were activated by triggers, such as thrombin, the adenosine molecules copackaged in the platelet dense granules would be secreted into the extracellular environment rapidly. According to previous works, adenosine molecules elicited from the thrombin-activated platelets (10^8^ platelets per ml) could reach 3–10 μM.^52,53^ To ascertain whether the secreted molecules were effective to activate the hydrogel substrate and control the adhesion of cells, the response of NIH 3T3 cells to ATP-producing platelets was examined. NIH 3T3 cells were first adhered on the aptamer-modified hydrogel surface, and then different amounts of platelets were co-cultured with them. To increase the response sensitivity, the incubation time of NIH 3T3 cells on the substrate was decreased to 2 h to avoid strong adhesion. As shown in the fluorescence image, without the addition of thrombin, the platelets have little influence on the adhesion of NIH 3T3 cells, and the NIH 3T3 cells spread well on the substrate (Fig. 5b). However, after the platelets were activated by thrombin, about 57% of NIH 3T3 cells were released from the surface within 20 min. As expected, the release rate of NIH 3T3 cells was correlated directly with the number of platelets co-cultured in the same substrate and the amount of thrombin added (Fig. 5c). In addition, we observed that the thrombin itself without the platelets could not induce obvious cell release (Fig. S8, ESI†). This result showed that thrombin was not the directing factor for cell release but rather was the inducer of endogenous ATP molecules. The DNA scaffold on hydrogel could recognize the platelet-secreted ATP in real time and then initiate the “duplex to aptamer” conformational switch. Subsequently, the NIH 3T3 cells adhered on the surface could be released.

## Discussion

Although ATP was used as the model signalling molecule here, this system can be programmed as a universal platform of cell microenvironment-responsive substrate. Since a wide range of aptamers have been developed, the stimulating factor could be extended to growth factors, metal ions, biomarker proteins, and even some pathogen and cancer cells. In addition, two different aptamers could be simultaneously modified on the surface to immobilize two kinds of cells. Then one signalling molecule could just activate its corresponding aptamer and stimulate the release of specific cells. This can therefore control the sequential release of multiple cells in distinct stages according to the signalling molecules. Furthermore, due to the flexibility of DNA in design and synthesis, two different aptamers could be integrated into one DNA scaffold to control the adhesion of cells in a highly organized fashion. Specially, the target cells could be designed to detach only when the two biological inputs were simultaneously present, and this mechanism was thus equivalent to a logical AND gate. Alternatively, the dynamic substrate could perform an “OR” logic gate operation, for which either of biological molecules could activate the release of target cells. This will allow accurate control over the cell–matrix interaction. In previous works, the controlled cell adhesion systems mainly focused on using physicochemical stimuli, such as pH, temperature, magnetic field, light, or electric field. In contrast, the hydrogel designed here could respond to specific biological signals within the cellular microenvironment and control cell adhesion behavior. As the signal molecules play an essential role in various biological regulation, the biologically responsive hydrogels will provide a more effective communication with the real biological system. They may ultimately enable us to mimic the dynamic features in complex cellular processes. This design can be used to manipulate cell behaviors for special applications, such as stem cell differentiation, tissue development, and cell-based therapy.

In the present work, aptamer was modified on the surface of hydrogel to modulate cells cultured on the top of the hydrogel. Further exploitation of this approach for 3D cell culture and manipulation will hold great promise for biological applications. Recently, 3D aptamer-hydrogels have been reported by using aptamer-containing DNA structures as crosslinker to assemble polymer networks.^54,55^ The aptamer domains in the hydrogel could bind the target, causing the dissociation of crosslinker and disassembly of hydrogel. If cells are encapsulated inside the aptamer-crosslinked hydrogel, the hydrogel can recognize the special signaling molecule in the cellular microenvironment, and then make a controlled phase transition to regulate the embedded cells. This provides the potential for biological signal-responsive control of the cellular microenvironment and cell behaviors in a 3D manner. For practical applications, one problem may be raised relating to the stability of the DNA scaffold modified on the hydrogel. Nucleic acid (especially RNA) is susceptible to nucleases in biological media, particularly in blood. In our present *in vitro* study, to avoid such potential issues, the incubation time of the DNA scaffold with cells was short and no serum was added in the incubation buffer. For further practical applications, this issue can also be solved by several solutions. With the developments of nucleic acid synthetic technology, increasing interesting research has been reported to protect nucleic acid against nuclease degradation.^56–58^ For example, the use of modified nucleosides, such as 2′-aminopyrimidine nucleosides, 2′-fluoropyrimidine nucleosides, 2′-*O*-methylpurine and 2′-*O*-methylpyrimidine nucleosides, significantly increased the resistance to nuclease. Some of these modified nucleic acids have been successfully adopted for *in vivo* study. Therefore, it is possible that dynamic hydrogels will provide potential for cell-based fundamental study and biomedical applications.

## Conclusions

The aptamer-based DNA scaffold could recognize biological signal in real time, and then dynamically control the presentation of bioligands on a hydrogel. This provided a flexible strategy to regulate the surface property of hydrogels as well as the behavior of cells on them. Both the exogenous and the surrounding cell-secreted ATP could activate the scaffold and tune the cell–substrate interactions. To the best of our knowledge, this is the first example of a dynamic hydrogel which can recognize intercellular signalling to control cell adhesion. With the extensive developments of aptamers, hydrogels could be readily designed to respond to multiple biological inputs. In addition, by combining different kinds of aptamers, this system could be further programmed to control multiple cells in a highly organized manner. The advantage of flexible design, convenient operation, and ready engineering makes aptamers very suitable for the design of complex signal-responsive scaffolds. Compared with the traditional substrate, the biochemically responsive hydrogel rewires the communication between biological event, hydrogel property, and cell behaviors. Future works may be developed to engineer hydrogels to elicit targeted cellular behavior in special biomedical applications, such as the controlled release of therapeutic stem cells with the biomarkers in inflamed or tumor tissues.

## Experimental

### Trigger cell release by ATP

After the adherence of cells on the aptamer-functionalized substrate, the substrate was examined for ATP-triggered cell release. A series of 300 μl HEPES buffer solution (5 mM HEPES, 10 mM MgCl_2_, 137 mM NaCl, pH = 7.4) containing different concentrations of ATP (0, 20, 100, 200, 500 μM, 1 mM) were added onto the substrates in a 48-well plate. They were gently shaken at 37 °C at 20 rpm for 2.5, 5, 7.5, 10, 15 or 20 min. After that, the ATP solutions were placed with the washing buffer (pH = 7.4). Then the substrates were further shaken at 80 rpm for 2 min to allow the released cells to be removed from the hydrogel surface. The cells remaining on the substrate were stained with AM dye and measured with a fluorescence microscope to quantify the cell number. To determine whether the stimulus process was biocompatible, the viability of released cells was assessed with live/dead assay. For this, the released cells were collected from the washing buffer by centrifugation at 1000 rpm for 5 min, and then propidium iodide (PI) and calcein (AM) dyes were added into the cell solution with a final concentration of 2 μM. After 15 min staining, the cells were rinsed with pH = 7.4 PBS buffer three times. The cell images were obtained by fluorescence microscopy. The released cells were also re-incubated on the glass side for 0, 12 and 48 h and then monitored with microscopy. The proliferation potential of released cells was further evaluated by MTT (methylthiazoletetrazolium) assay. After incubation in a cell culture plate for different times, the cells were treated with MTT solution for 4 h. Subsequently, the supernatant was discarded, followed by the addition of DMSO into each well. Then the optical density (OD) of the resulting solution was read at a wavelength of 570 nm. Proliferation index = *N*_*H*_/*N*_*H*=0_, where *N*_*H*_ = the cell number after incubation for *H* hour, *N*_*H*=0_ = the cell number at the beginning.

### Trigger cell release by ATP secreted from platelets

The platelets were isolated from whole blood.^59–61^ Approximately 20 ml of blood was collected into sterile heparinized vacutainer tubes. Then the whole blood was centrifuged immediately at 1200 rpm for 15 min at room temperature to obtain platelet-rich plasma (top fraction). The yellow platelet-rich plasma was transferred to a fresh conical tube carefully to avoid collecting any of the buffy coat. To obtain a platelet pellet, the platelet-rich plasma was centrifuged at 3500 rpm for 15 min at room temperature. The platelet pellet was then resuspended in PBS buffer (pH 7.4). The number of platelets was counted using a hemacytometer. For control of cell release by ATP secreted from the platelets, the NIH 3T3 cells were first adhered on the hydrogel surface in a 96-well plate for 2 hours. Then different amounts of platelets were loaded into the same well. After a brief incubation at 37 °C, the palates were activated by addition of 0.5 μl of thrombin.^52,62^ After gently shaking at 37 °C for 20 min, the hydrogel was washed with PBS buffer (pH = 7.4) and the cells on it were stained and imaged with a microscope.
