# Supplementary material for: Endogenous signalling control of cell adhesion by using aptamer functionalized biocompatible hydrogel
Source: Chem Sci. 2015 Aug 27;6(12):6762–8. doi: 10.1039/c5sc02565f (PMC5508704; doi:10.1039/c5sc02565f)
Supplement: Supplementary file 1 [file SC-006-C5SC02565F-s001.pdf]

Electronic Supplementary Material (ESI) for Chemical Science.
This journal is © The Royal Society of Chemistry 2015

Supporting Information

Endogenous Signalling Control of Cell Adhesion by Using Aptamer Functionalized Dynamic Hydrogel

Experimental Section

Reagents and Materials: 3-(Methacryloyloxy)propyltrimethoxysilane (MPS), propidium iodide (PI) and calcein (AM) dye were purchased from Aldrich. N-Hydroxysulfosucnimide sodium salt (sulfo-NHS), 1-[3-(dimethylamino)propyl]-3-ethylcarbodiimide hydrochloride (EDC), and fluorescein isothiocyanate (FITC) were obtained from Alfa Aesar. Alginate sodium, methacrylic anhydride, eosin Y, N.N'.-Disuccinimidyl Carbonate (DSC), and N-vinylpyrrolidone (NVP) were purchased from Aladdin (China). 4-Maleimidobutyric acid N-hydroxysuccinimide ester (GMBS) (97%) was obtained from Acros Organics. The nucleoside triphosphates (adenosine triphosphate (ATP), guanosine triphosphate (GTP), cytidine triphosphate (CTP) and thymidine triphosphate (TTP)) used in this paper were offered by Biotechnology Inc. (Shanghai, China). DNA sequences used were shown in Table S1. All oligonucleotides and the RGD peptide were synthesized by Sangon Biotechnology Co. (Shanghai, China). All other reagents were of analytical reagent grade and used as received. All aqueous solutions were prepared with nanopure water (18.2 MU, Milli-Q, Millipore).

Apparatus and Characterization: UV absorbance measurement was carried out on JASCO V-550 UV-vis spectrophotometer, equipped with a Peltier temperature control accessory. The DNA concentration was determined by measuring the absorbance at 260 nm at high temperature (95℃) by using a Carry 300 UV/Vis spectrophotometer connected to a thermal peltier controller. The extinction coefficient was calculated from mononucleotide and dinucleotide data by using the nearest-neighbor approximation. FT-IR characterization was carried out on a BRUKE Vertex 70 FT-IR spectrometer. Sample was thoroughly ground with exhaustively dried KBr. ^{1}H NMR (400 MHz) spectra were recorded using a Bruker Avance 400 NMR spectrometer using D_2O as the

solvent. Scanning electron microscopic (SEM) images were recorded using a Hitachi S-4800 Instrument (Japan).

Preparation of acrylamide-functionalized glass slide: Glass slides were cut into small pieces with a dimension of 4 mm × 4 mm. The glass slides were firstly cleaned by soaking them in Piranha solution (98% H_2SO_4/30% H_2O_2 = 3/1) overnight and subsequently rinsed with Milli-Q water. After drying in nitrogen stream, the clean glass slides were immersed into 5 mL of 4% 3-(methacryloyloxy)propyltrimethoxysilane (MPS) solution in ethanol for 4 h to introduce acrylamide group. The silanized glass slides were successively washed with pure ethanol and water. To cure the MPS layer, the glass slides were loaded in an oven for 60 minutes at 100 °C. The obtained glass slides were stored in a vacuum desiccator.

The modification of alginate with methacrylate groups: The methacrylated alginate was synthesized by reacting the secondary alcohols of sodium alginate with methacrylate groups.[1-5] To this end, methacrylic anhydride (20-fold excess) was added dropwise into a 1% w/v solution of alginate in deionized water. The pH of solution was adjusted to 8.0 with 5 M NaOH. After reacting for 48 h, the macromer solution was dialyzed (molecular weight cut-off=10 kDa) against deionized water for at least 3 days. The methacrylated alginate (MA-alginate) was obtained by lyophilization. The successful methacrylate modification was confirmed by comparing the ^1H NMR spectra (in D_2O) of alginate and methacrylate-modified alginate. For the methacrylate-modified alginate, new peaks at 1.8–2.0, 5.7–5.9, 6.1–6.3 ppm appeared, that were the methacrylate proton peaks.

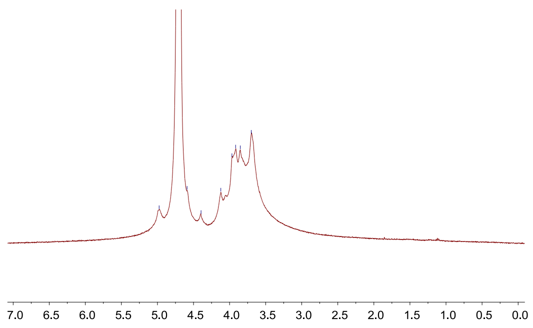

The ^1H NMR spectrum of alginate polymer (in D_2O).

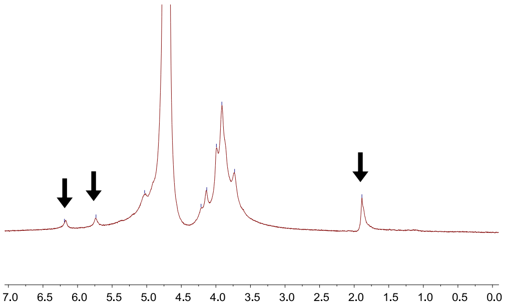

The ^1H NMR spectrum of methacrylate-modified alginate polymer (in D_2O). The new peaks δ= 6.19 ppm and 5.73 ppm were the methylene protons and the peak δ= 1.89 ppm was the methyl group peak.

Coating alginate hydrogel on glass surface: A thin layer of alginate hydrogel was synthesized on the silanized-functionalized glass surface. 4 μL of 5 mg/mL MA-alginate solution containing 10 μM eosin Y photoinitiator, 0.5 % (v/v) of N-vinylpyrrolidone (NVP), and 1.5 % (v/v) triethanolamine (TEA) was dropped on the previously obtained silanized glass slide.[6-8] A piece of clean coverslip was placed on the surface of alginate solution. Then the MA-alginate solution was crosslinked by exposure to visible-light source (200 Watt quartz halogen lamp) for 2 min. After removing the coverslip carefully, the alginate hydrogel-modified glass slides were obtained. The obtained substrates were rinsed with water thoroughly and then stored at 4 °C before use.

Modification of Apt-DNA on the hydrogel surface: The NH_2-Apt-DNA was modified on the hydrogel surface using standard carbodiimide chemistry. The hydrogel substrates were immersed into 1 mL of 2-(N-morpholino)-ethanesulfonic acid hydrate buffer solution (MES, 20 mM, pH 6.0) which contained 2 mg N-(3-dimethylaminopropyl)-N'-ethylcarbodiimide HCl (EDC-HCl) and 1 mg sulfo-N-hydroxysulfosuccinimide (sulfo-NHS). After activation of the carboxyl group of alginate hydrogel, the DNA-NH_2 (20 μL of 25 μM) was dropped on the surface. After incubation overnight, the substrates were washed by PBS buffer (pH 7.0) to remove the unbound DNA.

Coupling of FITC dye to NH_2-modified cDNA: For preparation of the fluorescein isothiocyanate (FITC)-modified DNA, the 5'-amino-DNA were firstly dissolved in phosphate buffer solution (10 mM, pH 7.0) with a final

concentration of 50 μM. Then, 2 μL of 6.25 mM FITC in DMSO were added into 50 μL of amino-DNA solution. The FITC with the isothiocyanate group could couple with the amino group of DNA. After reaction in dark for 8 hours, the excess dye was separated by dialysis through the molecularporous membrane tubing (MWCO: 2000, Spectrum Laboratories, Inc. US).[9] After the modification of FITC, the DNA exhibited emission peak at 520 nm.

The fabrication of FITC labeled substrate: The Apt-DNA-modified alginate hydrogel coating was incubated in a solution of 20 μM FITC-cDNA (100 mM phosphate, 1.5 M NaCl, 1 mM $MgCl_2$, pH=7.0) at 37 ^oC for 6 h to realize the DNA hybridization. The surface was then washed with PBS buffer (pH=7.0) and imaged using an Olympus BX-51 optical system microscope (Tokyo, Japan). For testing the ATP-responsive ability, the substrate modified with the hybridized DNA was further incubated with 0.5 mM ATP in the HEPES buffer solution (5 mM HEPES, 10 mM $MgCl_2$, 50 mM NaCl, pH=7.4) at 37 ^oC for 15 min. After washing, the substrate was characterized with microscope.

Cell culture: The NIH 3T3 mouse embryonic fibroblast cells (NIH 3T3 fibroblast) were cultured in Dulbecco's modified eagle medium (DMEM) supplemented with 10% fetal bovine serum and 1% Penicillin-Streptomycin at 37 ℃ in a humidified atmosphere of 5% CO_2. When the cells were grown to 80% confluency, they were detached from the flask by trypsin. The cells were collected by centrifugation for further use. The number of cells was counted with hemacytometer.

The fabrication of RGD modified hydrogel substrate for cell adhesion: The RGD was conjugated to cDNA according to previous works by using the bifunctional cross-linker.[10-16] The Apt-DNA modified alginate hydrogel coating was incubated in a solution of 20 μM RGD-cDNA (100 mM phosphate, 1.5 M NaCl, 1 mM $MgCl_2$, pH=7.0) at 37 ^oC for 6 h to realize the DNA hybridization. The surface was then washed with PBS buffer and incubated with 1×10^5 NIH 3T3 cells for 3 hours. The unbound cells were gently removed from the coatings by rinsing with buffer for 1 min. Cells were stained by AM fluorescent dye for 10 min and viewed using an Olympus BX-51 optical

system microscope (Tokyo, Japan). To assume the biocompatibility of hydrogel substrate, cells were further cultured on substrate for 24 hour, and then their viability was also assayed with live/dead cell staining. For this, the propidium iodide (PI) and calcein (AM) dyes were incubated with cells with a final concentration of 2 μM. After 15 min incubation, the cells were imaged under the microscope.

References

1 E. M. Chandler, C. M. Berglund, J. S. Lee, W. J. Polacheck, J. P. Gleghorn, B. J. Kirby, C. Fischbach, *Biotechnol. Bioeng.,* 2011, **108**, 1683.

2 S. J. Bidarra, C. C. Barrias, K. B. Fonseca, M. A. Barbosa, R. A. Soares, P. L. Granja, *Biomaterials* 2011, **32**, 7897.

3 K. B. Fonseca, D. B. Gomes, K. Lee, S. G. Santos, A. Sousa, E. A. Silva, D. J. Mooney, P. L. Granja, C. C. Barrias, *Biomacromolecules,* 2013, **15**, 380.

4 O. Jeon, K. H. Bouhadir, J. M. Mansour, E. Alsberg, *Biomaterials,* 2009, **30**, 2724.

5 K. Y. Lee, E. Alsberg, S. Hsiong, W. Comisar, J. Linderman, R. Ziff, D. Mooney, *Nano. Lett.,* 2004, **4**, 1501.

6 L. Degoricija, P. N. Bansal, S. H. M. Söntjens, N. S. Joshi, M. Takahashi, B. Snyder, M. W. Grinstaff, *Biomacromolecules,* 2008, **9**, 2863.

7 D. Nettles, T. P. Vail, M. Morgan, M. Grinstaff, L. Setton, *Ann.Biomed. Eng.,* 2004, **32**, 391.

8 C. L. Franco, J. Price, J. L. West, *Acta. Biomater.,* 2011, **7**, 3267.

9 O. I. Wilner, Y. Weizmann, R. Gill, O. Lioubashevski, R. Freeman, I. Willner, *Nat. Nano.,* 2009, **4**, 249.

10 B. M. G. Janssen, W. Engelen, M. Merkx, *ACS Synth. Biol.,* 2014, DOI: 10.1021/sb500278z.

11 V. Lapiene, F. Kukolka, K. Kiko, A. Arndt, C. M. Niemeyer, *Bioconjugate Chem.,* 2010, **21**, 921.

12 J. H. Lee, D. W. Domaille, J. N. Cha, *ACS Nano,* 2012, **6**, 5621.

13 J. Beuvelot, D. Portet, G. Lecollinet, M.-F. Moreau, M. F. Baslé, D. Chappard, H. Libouban, *J. Biomed. Mater. Res. B*., 2009, **90B**, 873.

14 L. Wu, J. Ren, X, Qu. *Nucleic Acids Res.,* 2014, **42**, e160.

15 M. You, R.-W. Wang, X. Zhang, Y. Chen, K. Wang, L. Peng, W. Tan, *ACS Nano,* 2011, **5**, 10090.

16 J. Michael, L. Schonzart, I. Israel, R. Beutner, D. Scharnweber, H. Worch, U. Hempel, B. Schwenzer, *Bioconjugate Chem.,* 2009, **20***,* 710.

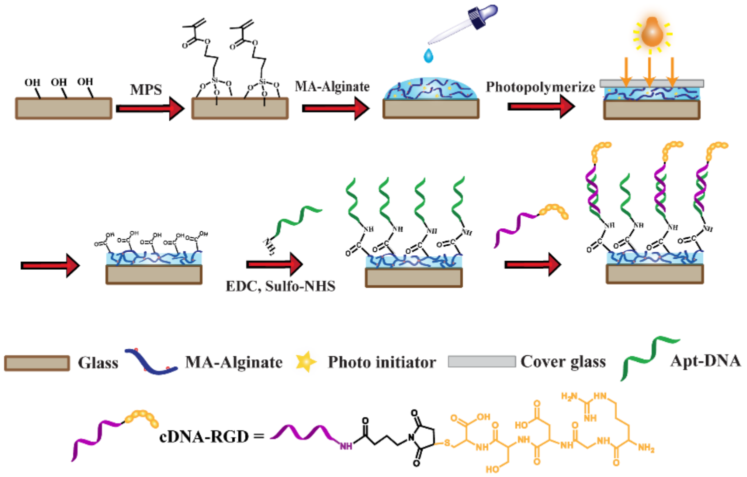

Scheme S1. The detail preparation process of signalling-sensitive substrate by using aptamer functionalized alginate hydrogel.

Table S1. The DNA sequences used in our experiments. The complementary region between ATP aptamer and its cDNA was highlighted with bold style.

The ATP aptamer DNA (Apt-DNA)	5′-NH_2-AA**A AAG AGA ACC TGG G**GG AGT ATT GCG GAG GAA GGT -3′
The complementary single-stranded DNA (cDNA)	5′-NH_2-AAA AA**C CCA GGT TCT CTT T**-3′
The ATP-insensitive random DNA (control DNA for ATP aptamer)	DNA1: 5′-GAG AGG AGA GAG AAG AGG AAG TTT TTT -NH_2-3′
The complementary DNA of ATP-insensitive random DNA	DNA2: 5′-CTT CCT CTT CTC TCT CCT CTC TTT TTT -NH_2-3′

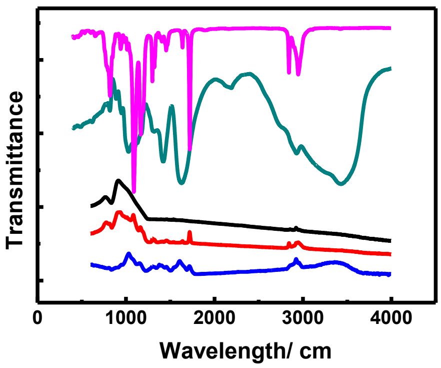

Fig. S1. Infrared spectra of the glass substrate (black line) and 3-(methacryloyloxy)propyltrimethoxysilane (MPS) modified glass substrate (red line) and the alginate hydrogel modified glass substrate (blue line). The infrared spectra of MPS (magenta line) and alginate polymer (green line) were also showed to confirm the successful modification of MPS and alginate hydrogel on the glass substrate.

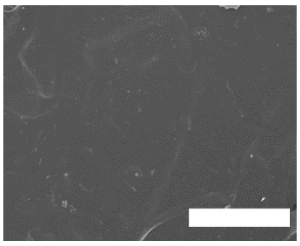

Fig. S2. Scanning electron micrograph of the alginate hydrogel modified surface. The scale bar was 50 µm.

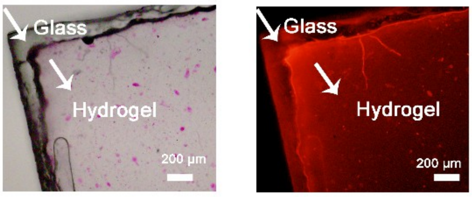

Fig. S3. The bright field image and corresponding fluorescence image of the alginate hydrogel coating. During the synthesis of hydrogel, rhodamine-B was encapsulated into the hydrogel to visualize it with fluorescence microscope.

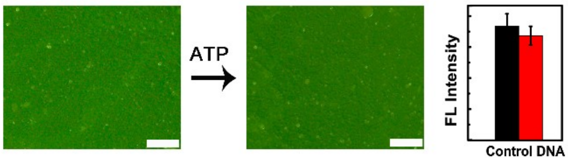

Fig. S4. The fluorescence images and the relative fluorescence intensity of FITC-functionalized control substrates without (left) and after (right) ATP treatment. The scale bars were 50 μm.

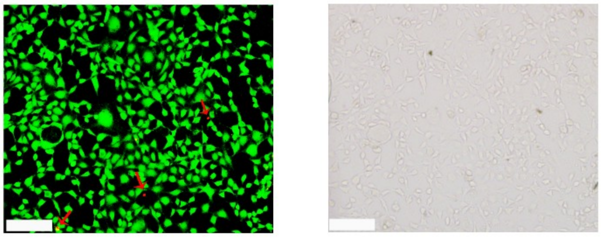

Fig. S5. The live (green)/dead (red) staining and corresponding bright field image of cells incubated on RGD-modified alginate hydrogel surface for 24 h. The scale bars were 100 μm.

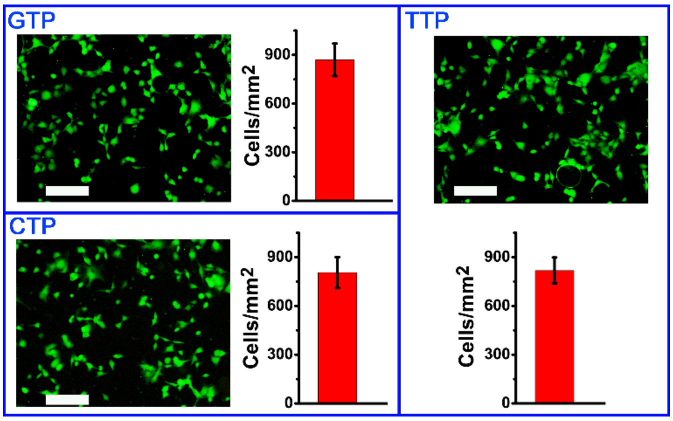

Fig. S6. The fluorescence images and quantitative number of cells on hydrogel substrate after the treatment of 0.5 mM of GTP, CTP and TTP. The scale bars were 50 μm.

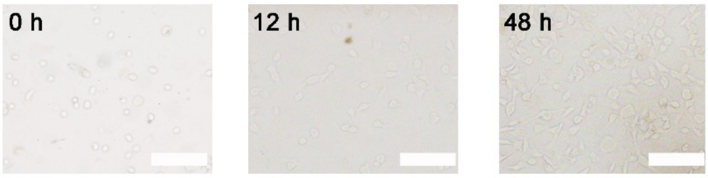

Fig. S7. The spreading and proliferation of the normal control cells after seeding on glass slide for 0 h, 12 h, or 48 h. The scale bars were 100 μm.

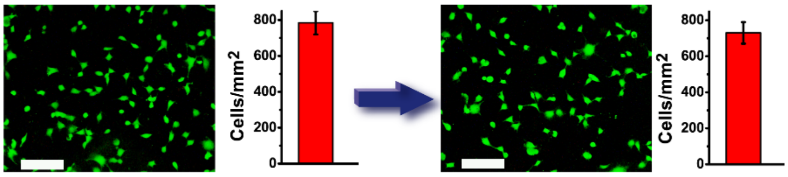

Fig. S8. The fluorescence images and quantitative number of NIH 3T3 cells before (left) and after (right) the addition of thrombin. The results showed that the thrombin itself without the platelets cells could not mediate the obvious release of NIH 3T3 cells. The scale bars were 50 μm.
